# The application of artificial intelligence in EUS

**DOI:** 10.1097/eus.0000000000000053

**Published:** 2024-04-10

**Authors:** Deyu Zhang, Chang Wu, Zhenghui Yang, Hua Yin, Yue Liu, Wanshun Li, Haojie Huang, Zhendong Jin

**Affiliations:** 1 Department of Gastroenterology, Changhai hospital, Naval Medical University, Shanghai 200433, China; 2 Department of Gastroenterology, General Hospital of Ningxia Medical University, Yinchuan 750004, Ningxia Hui Autonomous Region, China

**Keywords:** Artificial intelligence, EUS, Machine learning, Deep learning, Convolutional neural networks, Gastrointestinal disease, Pancreatic lesions

## Abstract

Artificial intelligence (AI) is an epoch-making technology, among which the 2 most advanced parts are machine learning and deep learning algorithms that have been further developed by machine learning, and it has been partially applied to assist EUS diagnosis. AI-assisted EUS diagnosis has been reported to have great value in the diagnosis of pancreatic tumors and chronic pancreatitis, gastrointestinal stromal tumors, esophageal early cancer, biliary tract, and liver lesions. The application of AI in EUS diagnosis still has some urgent problems to be solved. First, the development of sensitive AI diagnostic tools requires a large amount of high-quality training data. Second, there is overfitting and bias in the current AI algorithms, leading to poor diagnostic reliability. Third, the value of AI still needs to be determined in prospective studies. Fourth, the ethical risks of AI need to be considered and avoided.

## INTRODUCTION

Artificial intelligence (AI) was first mentioned in a 1950 article titled “Computational machinery and intelligence,” written by Turing, the father of computer science as well as AI. In that article, he asked the classic question: “Can a machine think?”^[1]^ Then, in 1956, several computer scientists gathered at the Dartmouth conference to propose the concept of “AI,” and they dreamed of using the computers that had just emerged to build complex machines that had the same essential properties as human intelligence. For the subsequent decades, AI had been mentioned in many top academic conferences and was constantly used in research laboratories. However, during the early stage of AI, because of imperfect algorithm theory and the limited computing power of hardware, the development of AI was very slow. In nearly a decade, thanks to the increase in data volume, the improvement in computing power, and the emergence of new machine learning algorithms (deep learning), research on AI began to make explosive progress, and since then, AI has been widely used in many fields, especially in the medical field.^[2–6]^

Endoscopic ultrasonography was introduced first by Wild and Reid in 1957 with a blind mechanical radial scanning probe introduced into the rectum.^[7]^ Endoscopic ultrasonography has undergone several improvements, such as a 360-degree scanning field and a progressive increase in ultrasound frequency and resolution. In the middle and late 1980s, not only did EUS link the anatomical wall and EUS but also recorded the depth of tumor penetration (T stage) and the metastasis (N stage) of local lymph nodes, and endoscopic ultrasonography clearly proved the superiority of EUS in lesions inside and near the gastrointestinal tract, as compared with other imaging modalities (such as computed tomography and magnetic resonance imaging). However, EUS cannot distinguish cancer from benign lesions (ie, pancreatic cancer from pancreatitis or inflammatory lymph nodes from metastases^[8]^). To solve this problem, a company developed EUS equipped with a biopsy channel, and in 1991, EUS-FNA of pancreatic lesions^[9]^ was successfully performed. To solve the problem of less tissue collected by EUS-FNA puncture, some scholars designed the side cutting and front cutting needles of the biopsy (EUS-FNB) and verified that the latter needles collected significantly more tissue.^[10–12]^ In addition, needle-based confocal laser microscopy, which enables real-time microscopy *in vivo* imaging of tissue surfaces, is used as an alternative to microbiopsy.^[13–17]^ Furthermore, EUS-guided procedures for peripancreatic fluid collections have been shown to be superior to percutaneous and surgical techniques in terms of morbidity, length of hospital stay, and costs.^[18]^

However, EUS is limited by the inherent characteristics of ultrasound imaging and higher requirements for endoscopic physicians, the diagnostic yield may drop in the beginners or less experienced operators, and EUS does carry a small but real risk of pancreatitis, infection, pancreatic duct leak, malignant seeding, hemorrhage, and even death.^[19]^ Moreover, most senior EUS experts are often concentrated in senior medical centers. As a result, with less experienced EUS physicians, EUS may lead to insufficient lesion detection or a misdiagnosis. Furthermore, fatigue and carelessness sometimes lead to a misdiagnosis of tumors even when specialists perform EUS.^[20]^ It can be seen that there are still many limitations in the clinical application of EUS, and data analysis means big data, which makes AI urgently needed to assist ultrasound endoscopists in diagnosis and treatment.

With the wider application of EUS in the clinic and the continuous development of AI, an increasing number of researchers are studying how to better apply AI technology to EUS for the diagnosis of related digestive tract diseases.^[21–24]^ This review mainly introduces the application and advanced research fields of artificial intelligence in EUS diagnosis.

## THE CONCEPT AND APPLICATION OF AI

AI has been described as “a branch of computer science that aims to create systems or methods that analyse information and allow the handling of complexity in a wide range of applications.”^[25]^ In other words, AI is a new technology in which researchers design and create relevant algorithms, process relevant data without manual assistance, and draw corresponding conclusions.

### Assisted in diagnosis and image recognition

Artificial intelligence can assist doctors in clinical diagnosis and image recognition. For example, a deep learning–based AI model has been reported to have similar performance to dermatologists in the classification of skin cancers.^[26]^ Alternatively, deep learning can use images of lung,^[27]^ prostate,^[28]^ brain^[29]^ tumors to predict patient survival and tumor mutations. Other studies point to the important role of AI in the identification of effects in breast cancer screening.^[30]^

### Personalized medicine and treatment

Artificial intelligence can realize precision medicine^[4]^ and treatment plans by analyzing information about the patient's genome, physiological indicators, and medical history.^[31–33]^ Different doctors and nurses have different diagnoses and treatments and may not have the same treatment plans for the same disease, and different patients need to have their own personalized treatment plans. Taking the opportunity that AI offers, an AI-assisted medical security system will enable all doctors to practice at the same level of expertise as the best teams of doctors and share other data in addition to patient privacy risks on different medical platforms, enabling both medical staff and patients to achieve the best results.^[34]^

However, tasks that cannot be performed by machines because of the need for emotional intelligence, such as asking patients careful questions to detect more subtle symptoms and building trust by building personal relationships using human intuition, remain unique qualifications of physicians, and these can guide the implementation of future computationally optimized diagnosis and treatment plans.^[35]^

### Medical management and data analysis

Artificial intelligence can process large amounts of medical data and use machine learning algorithms for data analysis to support medical decision making and management. Some scholars believe that big data can improve diabetes care by establishing a large system, and by combining the information of the patient with diabetes with the big data, the health care professional and the health care system can use AI to provide accurate care of diabetic patients through data processing.^[36]^

The US Food and Drug Administration has approved IDx-DR, a device that uses AI algorithms to analyze digital retinal images and help with early detection of retinopathy.^[37,38]^ The American Diabetes Association has approved the use of autonomous AI to detect diabetic retinopathy and macular edema.^[39]^

### Robot-assisted surgery

Robotic surgical techniques are assisted surgical techniques based on AI.^[40]^ Unlike public expectations, the development and adoption of autonomous robots in medical interventions are much slower. For decades, robotic surgery has been synonymous with robot-assisted surgery, which promotes surgical procedures and makes motion smoother than human-accessible motion, but it still requires motion control by the surgeon.^[41]^

For instance, in the US Food and Drug Administration–approved da Vinci surgical system for minimally invasive operations, surgeons operate the robot from a console.^[42]^ Such systems are designed to translate the surgeon's hand movements into the movements of instruments inside the patient and are therefore not autonomous. However, surgeons have also made a breakthrough. Suturing is one of the most common procedures during surgery, and therefore, autonomous knotting robots have been developed.^[41]^ The supervised autonomous robotic system for suturing an intestinal anastomosis showed superior *in vivo* suture quality compared with that of surgeons in a laboratory setting.^[43,44]^

With the continuing development of preprogrammed, image-guided, and teleoperated surgical robots, more robot-assisted or automated intervention methods are expected to be incorporated into surgical practice.^[45–47]^ A balance needs to be struck between patient rights, commercial value, and the needs of AI researchers to provide the big data needed to build deep learning models as precursors to autonomous robots.^[23]^ Artificial intelligence researchers predict that AI-powered technologies will outperform humans at surgery by 2053.^[35]^

In general, the application of AI in the medical field can provide doctors with more accurate, efficient, and safe diagnosis and treatment solutions and provide a better medical experience and treatment effect for patients. With the continuous development and innovation of AI technology, it is believed that it will bring more opportunities and challenges to the medical field.

## THE ALGORITHM INTRODUCTION OF AI

The most basic practice of machine learning is to use algorithms to parse data, learn from it, and then make decisions and predictions about real-world events.^[48]^ Unlike traditional hard-coded software programs that solve specific tasks, machine learning “trains” with large amounts of data and learns how to complete tasks from the data through various algorithms.

**Figure 1:**
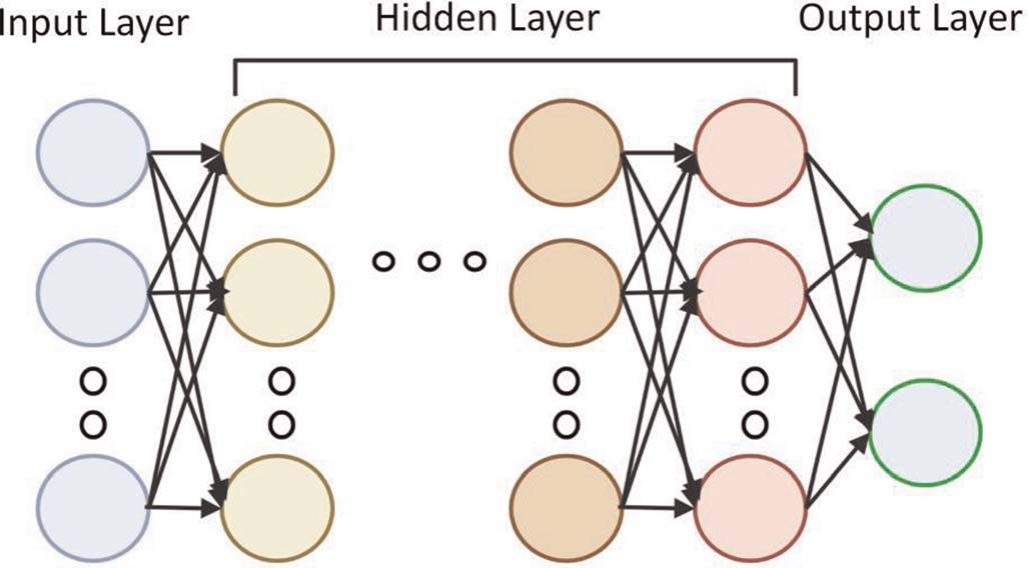
Neural network principle: neurons are connected in series or in parallel, and the neural network consists of an input layer, several hidden layers, and an output layer. Created by MS Office (https://www.microsoft.com/zh-cn/).

Machine learning is directly derived from the early field of AI, which includes multiple techniques, such as support vector machines (SVMs), decision trees, factor machines, logistic regression analysis, and neural networks.^[48,49]^ A neural network is a machine learning technique based on the use of multiple neurons.^[50]^ Each neuron converts the input data into the output data by applying a weight to the input data (thus adding a bias) and passing it to the activation function. Neurons can be connected in series or in parallel, and a neural network consists of an input layer, several hidden layers, and an output layer [Figure 1].

Given the commonalities shared between statistical and machine learning techniques, the boundary between the 2 may seem fuzzy or ill-defined. One way to describe these methods is to consider their primary objectives. Both are used to infer results, but unlike statistics, in which the goal is to understand the relationship between variables, machine learning is the result of predicting all variables, even if they are nonlinear regressions, and the relationship between variables is not important. Among them, machine learning can be simply divided into supervised (labeled data) and unsupervised (unmarked data) learning technology. Supervised learning refers to the technique of training models on a series of inputs (or features) associated with known results, whereas unsupervised technology is exploratory to discover undefined patterns or clusters in datasets and does not involve predefined results.^[51]^

**Figure 2:**
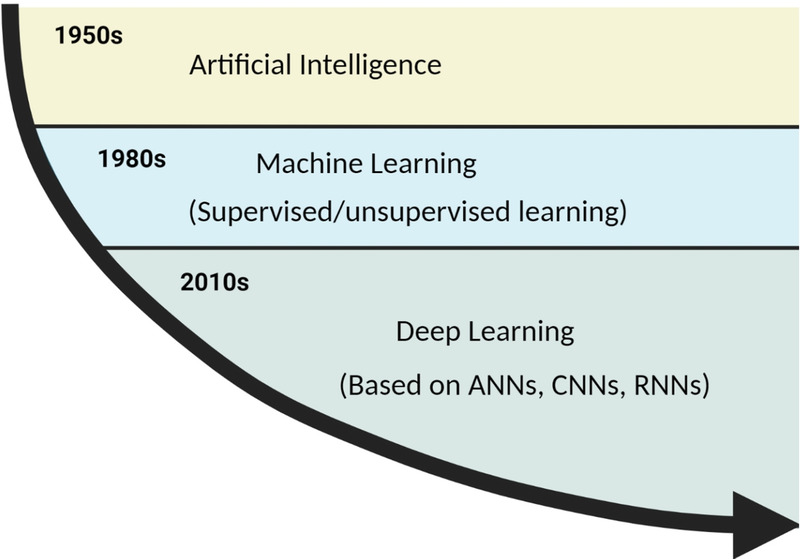
Relationship between AI, machine learning, deep learning algorithms. AI: artificial intelligence. Created by the Biorender (https://www.biorender.com/).

An artificial neural network is a set of algorithms used for machine learning. Its appearance failed to set off the stormy waves of AI research; until in recent years, it returned to the public view under a new name—deep artificial network (Deep Artificial Networks, Deep Learning), and people re-recognized and valued it. Some of the greatest successes of deep learning have been in the field of computer vision. Computer vision focuses on image and video understanding and deals with tasks such as object classification, detection, and segmentation. Overall, machine learning is a way to realize AI, and deep learning is a technology to realize machine learning. The relationship between algorithms such as AI, machine learning, and deep learning is shown in Figure 2.

Convolutional neural networks (CNNs), a type of deep learning algorithm designed to process images, have grown to be central in this field. The CNN can be trained to classify images, segment regions of interest, and even detect specific features, such as tumors or lesions. An example of a CNN-based algorithm for medical image analysis is DeepLesion.^[52,53]^ It is a dataset that uses CNN to detect and locate lesions on computed tomographic scans with an accuracy of 81.1% and can be used in lesion detection, lesion classification, lesion segmentation, lesion retrieval, and lesion growth analysis.^[54]^

Recurrent neural network (RNN) is another deep learning algorithm that can be used for medical image analysis. Recurrent neural network is well suited for sequence data such as time series or sequence images. In medical imaging, RNNs can be used to analyze video sequences or multiframe images, where the information from the previous frame is important for the analysis of the current frame. An example of an RNN-based medical image analysis algorithm is the convolutional LSTM network (ConvLSTM). ConvLSTM is a deep learning architecture combining the spatial processing of the CNN with the temporal processing of the RNN. It has been used in video segmentation and disease diagnosis in medical imaging.^[55–58]^

In addition, there are many other types of machine learning algorithms. For example, SVMs^[59,60]^ are machine learning algorithms that can be used for medical image analysis. Support vector machines are binary classifiers that learn the decision boundary between 2 classes of data. In medical imaging, SVMs can be trained to distinguish healthy from diseased tissues or to detect specific features, such as tumors or lesions. An example of a medical image analysis algorithm based on SVMs is an SVM classifier (SVM-RBF) with radial basis functions.^[61]^

This algorithm has been applied to breast cancer diagnosis on images of nuclei sampled in breast masses and is significantly superior to regularized regression using generalized linear models and artificial neural networks.^[51]^

In conclusion, the AI-assisted diagnosis of medical images is a rapidly evolving field with the potential to significantly improve health care. The algorithms mentioned in this article are presented in Table 1. These algorithms can help doctors make more accurate diagnoses, thus improving the treatment of patients. With the continuous improvement of AI technology, we look forward to seeing the advent of more advanced algorithms to assist physicians in more effectively and accurately diagnosing the vast number of medical images, including EUS images.

**Table 1:** Summary of AI algorithms.

Algorithm	Function	Relationship	Application
Machine learning (ML)	Analyze the data to make decisions and predictions	From artificial intelligence	Through large lot of data “training” to learn how to complete the data through various algorithms
Deep learning (DL)	Classify, detecting, and segment objects	A technique for implementing machine learning	Computer vision
Convolutional neural network (CNN)	Classify images, segment regions and detect specific features	Classical algorithms in deep learning	DeepLesion
Recurrent neural network (RNN)	Analyze video sequences or multiple-frame images	Deep learning algorithms that focus on sequence data	ConvLSTM
Support vector machine (SVM)	A binary classifier for learning the decision boundary between 2 classes of data	Machine learning algorithms for medical images	SVM-RBF

## Application of AI in Endoscopic Diagnosis of Pancreatic Lesions

Artificial intelligence was first applied in the field of cancer in the diagnosis of endoscopic ultrasonography. Multiple previous studies of AI diagnosis in pancreatic ductal adenocarcinoma have been reported. For example, Zhang et al.^[62]^ used the SVM algorithm in machine learning to distinguish pancreatic ductal adenocarcinoma (PDAC) from normal tissue, identified 29 features combined with EUS images, and then built predictive models and trained repeatedly, resulting in a classification accuracy of 99.07% and a sensitivity of 97.98%. In the presence of chronic pancreatitis (CP), the diagnostic accuracy is significantly reduced because of endoscopic US and EUS-FNA/B because tumor and inflammatory changes generally have similar image changes. Meanwhile, EUS-FNA/B also relies heavily on the accurate localization of the region of interest based on the interpretation of the EUS image, and it has a low diagnostic yield, even when subjectively assessed by an experienced endoscopist. Das et al.^[63]^ retrospectively included 22 healthy patients after pancreatic EUS-FNA puncture, 12 patients with CP, and 22 patients with pancreatic cancer. Based on the 11 features extracted from EUS images, a machine algorithm was used to distinguish pancreatic cancer, CP, and normal pancreatic tissue, and the diagnostic model had a sensitivity of 93% and a specificity of 92%. Therefore, the researchers proposed that the performance characteristics of AI-assisted EUS diagnosis are comparable to those of EUS-FNA, and noninvasive diagnosis can be achieved in the future.

Săftoiu et al.^[64]^ prospectively included patients with endoscopic pancreatic nodules, including 112 with pancreatic cancer and 55 with CP, for the differential diagnosis of pancreatic ductal carcinoma and focal CP using a CNN algorithm in deep learning. In the architecture of this study, deep learning is implemented in 2 stages, namely, the feature extraction stage and training stage. In the first stage, endoscopic ultrasonography of the pancreatic tumors was performed. Histogram analysis was performed on endoscopic elastography images, and several features were extracted. In the second phase, several features extracted in the first phase are used as input to the deep learning algorithm to judge pancreatic cancer and CP.^[65]^ After repeated training, the final model yielded a sensitivity of 94.64%, a specificity of 94.44%, a positive predictive value of 97.24%, and a negative predictive value of 89.47%. Therefore, the use of AI combined with EUS images can distinguish pancreatic cancer and CP cases, greatly improving the diagnostic accuracy and reducing the rate of misdiagnosis and missed diagnosis.

Pancreatic lesions mainly include not only tumor lesions,^[66,67]^ such as PDAC, pancreatic adenosquamous cell carcinoma, acinar cell carcinoma, metastatic pancreatic cancer, neuroendocrine carcinoma, neuroendocrine tumor, and real pseudopapillary tumor, but also some nonneoplastic lesions, such as CP and autoimmune pancreatitis. The diagnosis of pancreatic lesions as cancer or noncancer is important for the patient to undergo surgery and further treatment options. Recently, Kuwahara et al.^[23]^ conducted a retrospective study of 933 patients with pancreatic lesions using the advanced deep convolution generative antagonistic network (DCGAN) algorithm with EUS images to establish a deep learning model that could judge pancreatic lesions as cancerous or noncancerous based on EUS images. This study was conducted in 2 stages. The first stage extracted the predicted value of each static EUS image output by the deep learning algorithm and evaluated the diagnostic performance of the deep learning model at the image level. In the second stage, the predicted values of comprehensive continuous images from the video images were extracted, and the median values of all still images in the cohort were verified and tested at the patient level. Then, the diagnostic performance of this deep learning model was evaluated. The final result showed that the deep learning model had an accuracy of approximately 90% at both the image and patient levels. The use of the DCGAN algorithm can improve the imbalance caused by the input dataset, which ultimately enables the diagnostic model produced by the deep learning algorithm combined with EUS images to greatly improve the accuracy of tumor diagnosis. This deep learning model uses EUS images of almost all types of pancreatic masses and achieves a reliable diagnosis of tumors or nontumors. In addition, Tonozuka et al.^[20]^ developed the original computer-aided diagnosis system of CNN using endoscopic ultrasonography images, reported its carcinoma of the pancreas detection capability, and used control images from CP and necrotizing pancreatitis patients as a preliminary study to analyze whether the EUS-CNN algorithm model could correctly identify pancreatic masses. The CNN algorithm inputs 139 patients, including 76 pancreatitis, 34 CP, and 29 necrotizing pancreatitis patients, for a total of 88,320 images after training and 10-fold cross-validation and independent testing. Finally, its sensitivity was 92.4%, and its specificity was 84.1%.

An intraductal papillary mucinous tumor (IPMN) is a precursor lesion in pancreatic cancer.^[68,69]^ Therefore, early detection of IPMN and prediction of whether it has an increasing risk of malignancy are crucial. In this field, Takamichi Kuwahara et al.^[70]^ reported 206 IPMN patients with surgical confirmation using endoscopic IPMN-related images as input data from a deep learning algorithm. Based on the pathological diagnosis after resection, these IPMN patients were classified as benign IPMN (pathology revealed low- and middle-grade dysplasia) and malignant IPMN by IPMN (pathology showed high-grade dysplasia and invasive carcinoma). Later, it was predicted according to endoscopic IPMN images and compared with the pathological results. The final result was that the sensitivity, specificity, and accuracy of malignant IPMN were 95.7%, 92.6%, and 94.0%, respectively, far exceeding the human diagnostic accuracy of 56.0%. That study revealed that deep learning–based AI algorithms may be a more accurate and objective method to diagnose IPMN malignancies than human diagnosis and conventional EUS features.

## APPLICATION OF AI IN EUS FOR THE DIAGNOSIS OF GASTROINTESTINAL STROMAL TUMORS

Gastrointestinal stromal tumors (GISTs) and gastrointestinal leiomyomas (GILs) are the most common subepithelial lesions (SELs). Gastrointestinal stromal tumors occur most frequently in the stomach, accounting for approximately 60% to 70% of cases, whereas the percent of small intestinal GISTs is approximately 20% to 30%, the percent of colorectal GISTs is approximately 5%, and the least is less than 5%.^[71]^ However, all GISTs are considered to have a certain degree of malignant potential, so early diagnosis and timely treatment are very important. EUS is helpful to diagnose SELs. The classic EUS image characteristics of GISTs are low dark echo appearance, round or oval, located in the fourth ultrasonic wall layer and corresponding to the muscularis propria. Although large or malignant stromal tumors can show irregular outlines, the tumor borders are usually smooth and clear, whereas the tissue can be heterogeneous or homogenous, with the mass occasionally possessing characteristics of echogenic foci, cystic spaces, or ulcers.^[72]^ Gastrointestinal leiomyomas are benign tumors distributed throughout the gastrointestinal tract, mostly occurring in the esophagus, esophagogastric junction, and stomach. Their EUS images are mostly uniform hypoechoic lesions, often located in the second or fourth ultrasound wall layer.^[73]^ Although it is possible to diagnose both at the pathological level by using fine needle aspiration biopsy, EUS-FNA still has limitations because of the risk and uncertainty of puncture and the lack of easy manipulation of smaller lesions.^[74]^ Relevant studies have combined AI technology with EUS to distinguish and diagnose GIST and GIL by a noninvasive method. Yang et al.^[75]^ designed an AI system based on EUS images of patients with GIST or GIL. They further developed and retrospectively evaluated this AI system by collecting information on EUS images of patients with these diseases from multiple centers. This system is used when endoscopists judge SELs as GISTs or GILs. The AI system in this study was developed using 10,439 EUS images from 752 GIST or GIL patients. Finally, the AI system was applied to a multicenter prospective diagnostic trial to explore whether the joint diagnosis of the endoscopist and the AI system could distinguish between GISTs and GILs at the clinical level. As a final finding, in the prospective trial, 132 subjects in 508 consecutive subjects were diagnosed histologically (36 GISTs, 44 GILs, and 52 other types of SELs). Through combined diagnosis (AI-assisted endoscopists), the accuracy of the endoscopists who diagnosed 80 patients with GISTs or GILs increased from 73.8% to 88.8%, and the total diagnostic accuracy was significantly improved.^[75]^

Kim and others^[76]^ have also developed a CNN-assisted diagnostic system to analyze EUS images of GISTs to distinguish GISTs that are difficult to distinguish from benign tumors (such as leiomyomas and schwannomas). First, EUS images of gastric GISTs, leiomyoma, and schwannoma were screened, and these lesions had been organized and pathologically confirmed by surgical or endoscopic resection and/or EUS-FNB, excluding blurred and poor-quality images. A total of 587 images from 179 gastric tumors were screened as a training image dataset, including 428 images from 125 GISTs, 91 images from 33 leiomyomas, and 68 images from 21 schwannomas. A total of 212 images from 69 gastric tumors were also collated as an independent test dataset, including 106 images of 32 GISTs, 60 images of 23 leiomyomas, and 46 images of 14 schwannomas. Then, a CNN algorithm model consisting of 5 layers was built. The output layer was first divided into 2 categories of tumors: GISTs and non-GISTs. In the non-GIST tumor group, the output layer was further subdivided into schwannomas and leiomyomas. For the final constructed CNN system, the sensitivity, specificity, positive predictive value, negative predictive value, and accuracy for distinguishing GIST tumors were 79.0%, 78.0%, 76.3%, and 80.5%, respectively, whereas the sensitivity, specificity, positive predictive value, negative predictive value, and accuracy were 89.3%, 80.6%, 82.1%, 88.4%, and 85.0%, respectively. Based on the obtained data, the investigators believe that the CNN system will show high accuracy in diagnosing GISTs on EUS images and can assist in the endoscopic diagnosis of GISTs in current clinical practice.

Hirai et al.^[24]^ collected EUS images of pathologically confirmed SELs of the upper digestive tract in 12 hospitals, including GISTs, leiomyoma, schwannoma, neuroendocrine tumor, and ectopic pancreas. Later, a random sampling method was used to divide the acquired images into a ratio of 4:1 (the dataset is used to develop and train the designed AI algorithm system) and a test dataset (the dataset is used to test the AI system). The study collected a total of 16,110 images from 631 cases for both datasets. The final results showed the accuracy of the AI system for five types of GISTs, leiomyoma, schwannoma, neuroendocrine tumor, and ectopic pancreas, which was significantly higher than that of all endoscopists. At the same time, the sensitivity, specificity, and accuracy of the AI system designed in the study to identify GISTs and non-GISTs were 98.8%, 67.6%, and 89.3%, respectively, which were also higher than those of endoscopists.

## APPLICATION OF AI IN ENDOSCOPIC DIAGNOSIS OF ESOPHAGEAL CANCER

At present, common esophageal lesions include submucosal tumors (including leiomyomas, stromal tumors, and lipomas), precancerous lesions, and esophageal cancer. Among them, esophageal cancer is one of the most common cancers, and it is also one of the most common causes of death. The early stage of esophageal cancer has no obvious abnormal symptoms; the most important symptoms in the advanced stage are progressive dysphagia and weight loss, which will make surgical resection necessary, and it often has a poor prognosis. However, with the gradual development of endoscopic technology, an increasing number of early esophageal cancer or esophageal precancerous lesions have been found, and through endoscopic resection, the patient's prognosis is also satisfying.^[20,77,78]^ Therefore, it is particularly important to improve the detection rate and diagnosis rate of early esophageal cancer so that patients can receive treatment at the early stage.^[79]^ Previous studies have revealed that the deep evaluation of the primary tumor extension to the esophageal wall and surrounding tissues by EUS can assist in the staging of esophageal cancer.^[80,81]^ However, EUS images also have some problems, such as low-resolution, blurred image artifacts and poor exposure to image quality.^[82,83]^

Wang and other researchers^[84]^ compared conventional endoscopy and AI algorithm-combined EUS. Comparing the endoscopic and ultrasonic images of real-time diagnosis characteristics combined with endoscopic detection and pathological results, they evaluated the AI algorithm combined EUS for the diagnostic value of early esophageal cancer and precancerous lesions. Through screening, 80 patients who met the standard were selected and randomly divided into 3 groups: 2 groups of EUS images based on the AI algorithm, which were then divided into a cascade region CNN (RCNN) model algorithm group and a traditional convolution neural network model algorithm group, and 1 group of EUS (control group). That study showed that the AI algorithm of ultrasonic images was effective, and the detection performance was better than endoscopic detection, which greatly reduced the detection time. The study calculated that the detection rates of the traditional CNN model, cascade RCNN model, and EUS alone were 56.3% (45 of 80), 88.8% (71 of 80), and 44.1% (35 of 80), respectively. The sensitivity, specificity, positive predictive value, and negative predictive value of the cascade RCNN model were all higher than those of the CNN model and EUS alone, providing a reference for the differential diagnosis of early upper digestive tract cancer and other digestive tract tumors.

A CNN algorithm system for automatically identifying the infiltration depth and origin of esophageal lesions was similarly developed by Liu et al.^[85]^ A total of 1670 EUS images collected for the study were used to train and validate the CNN system. The overall accuracy of the CNN system was 82.49%, with a sensitivity of 80.23% and a specificity of 90.56%. That study was the first to identify esophageal EUS images through deep learning, and a CNN algorithm was developed that can automatically identify the depth of invasion and lesion origin of esophageal submucosal tumors and can classify such tumors to achieve good accuracy.

Currently, preoperative staging of Barrett-related esophageal adenocarcinoma is the main criterion for determining the subsequent treatment strategy, and endoscopic ultrasonography is an important diagnostic method for preoperative staging. Related studies have shown that AI can assist in the diagnosis and staging of EUS and optimize treatment. Knabe et al.^[86]^ developed an AI system–assisted ultrasound endoscopy to stage Barrett-related esophageal adenocarcinoma. A total of 1020 images (at least 1 per patient, up to 3) from 577 Barrett adenocarcinoma patients were selected for CNN training and internal validation. A total of 821 images were selected to train the model, and 199 images were finally used to validate the model. The final results showed that the developed AI model had higher accuracy, sensitivity, and specificity in identifying benign Barrett mucosa lesions, Barrett mucosa carcinoma in situ, early Barrett esophageal progression carcinoma, and advanced Barrett progression carcinoma. The overall diagnostic accuracy rate was 73%.

## APPLICATION OF AI IN ENDOSCOPIC DIAGNOSIS OF LIVER AND BILIARY DISEASES

EUS is important for the diagnosis of biliary-related diseases,^[87–89]^ such as choledochthiasis, biliary obstruction, ampullary cancer, and cholangiocarcinoma, and AI also has unique value in the diagnosis of assisted EUS bile duct scans. Yao and other researchers^[90]^ have built a deep learning–based system called BP MASTER for real-time identification and bile duct labeling in EUS. The system in this study integrates 4 deep CNN (deep CNN, DCNN) algorithm models that have 2 functions: one is to locate the location of the ultrasound probe to provide corresponding operating instructions for the physician, and the other is to mark the bile duct and provide bile duct diameter measurements for endoscopists. DCNN 1 is used to filter out the gastroscope images and input the ultrasound images to DCNN 2. DCNN 2 divides the ultrasound images into standard and nonstandard categories and inputs the standard images into DCNN 3. DCNN 3 is used to identify the position of bile ducts, and DCNN 4 is used for segmentation and annotation of bile ducts. In this study, 2000 ordinary gastroscope images and 2000 EUS images were applied, and the BP MASTER system was obtained through strict testing. DCNN 1 classified gastroscope images and ultrasound images with 100% accuracy. In the standard and nonstandard image classification, DCNN 2 achieved an accuracy of 87.4%. However, for DCNN 3, the accuracy was 93.3%. For the segmentation performance for the bile ducts, the DCNN 4 segmentation was 77%. In summary, this BP MASTER system can identify the standard location of the bile duct scan, remind the endoscopist of the missed part, and guide the physician accordingly. At the same time, the system can also segment the bile duct with high accuracy, automatically measure the bile duct diameter, simplify the operation of the endoscopist, and help evaluate the dilated and narrow bile duct.

Gallbladder polypoid lesions are abnormal bulges of the gallbladder wall into the gallbladder cavity and have various pathological types. Currently, EUS is considered to be superior to conventional ultrasound in gallbladder examination, which can improve the differentiation of neoplastic gallbladder polyps and contribute to the staging of gallbladder cancer. The Jae Hee Cho research team developed an AI-assisted endoscopic diagnostic system with the ResNeT50 structure. A cohort of 1039 EUS images (including EUS images of 836 gallbladder polyps and 203 gallstones) were used for AI training, internal validation, and testing. Finally, the diagnostic performance was validated using an external validation cohort of 83 patients and compared with that of professional endoscopists. That study found that the accuracy of the diagnosis (65.3%) was between intermediate endoscopist (66.7%) and expert endoscopist (77.5%). The newly developed EUS combined with the AI diagnostic model of the research team showed good performance in the diagnosis of neoplastic gallbladder polyps and gallbladder adenocarcinoma, which is as good as that performed by endoscopists. This study reveals the broad prospect of AI combined with EUS technology in the diagnosis of gallbladder polyp diseases. However, at the same time, the relatively insufficient sample size may lead to a lack of AI training, so there are some limitations.^[91]^

In recent years, the role of EUS has become more important with emerging applications in the diagnosis and treatment of hepatology.^[92,93]^ EUS is considered a valuable tool for monitoring liver disease and complications by clear, real-time liver imaging.^[94,95]^ In the field of liver tumors, EUS has become an important tool for identifying, characterizing, and staging primary and malignant liver tumors.^[96]^ Focal liver lesions (FLLs) are an important concept in liver disease. Focal liver lesion includes not only malignant liver lesions but also solid and cystic benign lesions of the liver, such as hepatic hemangioma, focal nodular hyperplasia, hepatic adenoma, and liver cysts.^[97]^ Accurate discrimination between benign and malignant FLLs is key to optimizing the treatment of patients with possible primary liver cancer or metastatic tumors of the liver. In this field, Marya et al.^[98]^ developed a novel CNN model based on EUS to identify and classify FLLs. The study first reviewed a prospective EUS database that included cases of FLL visualized and sampled by EUS. Relevant static images and videos of the liver parenchyma and FLL were extracted. Patient data were then randomly assigned for CNN model training and testing. After the final model was created, an analysis was performed to evaluate the ability of the CNN model based on EUS images to independently identify FLL and the ability of the CNN model to identify benign and malignant FLL. That study used a total of 210,685 EUS images from 256 patients to train, validate, and test the CNN model. By analyzing this EUS-based CNN model, the FLLs in 92.0% of the EUS datasets were successfully located. When evaluating any random still images extracted from video or physician-captured images, the AI model was 90% sensitive and 71% specific for malignant FLL classification. When the full-length video dataset was evaluated with this model, its sensitivity was 100%, and its specificity was 80% for malignant FLL classification. However, one defect of endoscopic ultrasonography is the inability to obtain a complete evaluation of the right lobe of the liver, so the FLL in the right lobe may not have been fully visualized. In summary, that study demonstrated the accuracy, simplicity, and rapidity of identifying and classifying FLL based on endoscopic CNN model training.

## DISCUSSION

With the rapid development of AI technology in recent years, the diagnosis method of AI-assisted ultrasonic endoscopic imaging has shown vigorous vitality, liberating clinical ultrasound endoscopic physicians from heavy diagnostic work, and has been frequently used in liver biliary benign and malignant diseases, esophageal cancer, pancreatic benign and malignant diseases, and the identification of GISTs. With the development of AI algorithms and interventional ultrasound technology, in the future, AI can be further integrated with other examination means to accurately identify lesion properties and then provide treatment strategies. Artificial intelligefnce is also expected to provide accurate guidance or endoscopic ultrasonic interventional therapy. Examples of the endoscopic application of AI mentioned in this article are presented in Table 2.

**Table 2:** Summary of the application of artificial intelligence in EUS

Disease	Algorithm	Application	Object	Conclusion
Pancreatic lesions	SVM algorithm	Distinguish between PDAC and normal tissues	Combining EUS images to identify 29 features	Accuracy: 99.07%; sensitivity: 97.98%
	Machine algorithm	Distinguish between pancreatic cancer, chronic pancreatitis, and normal pancreatic tissue	Retrospectively included 22 healthy patients after pancreatic EUS-FNA puncture, 12 patients with chronic pancreatitis and 22 patients with pancreatic cancer	Sensitivity: 93%; specificity: 92%
	CNN algorithm	Differential diagnosis of pancreatic ductal carcinoma and focal chronic pancreatitis	Image data from 112 pancreatic cancers and 55 patients with chronic pancreatitis that were prospectively included	Sensitivity: 94.64%; specificity 94.44%; positive predictive value: 97.24%; negative predictive value: 89.47%
	Deep convolutional generative adversarial network (DCGAN)	The pancreatic lesions judged as cancerous or noncancerous based on EUS images	A retrospective study performed on 933 patients with pancreatic lesions with EUS images	About 90% accuracy at both the image and patient levels
	The original computer-aided diagnostic system of the CNN	To detect pancreatic cancer and to distinguish between chronic pancreatitis and necrotizing pancreatitis	76 patients with pancreatic cancer, 34 with chronic pancreatitis, and 29 with necrotizing pancreatitis.	Sensitivity: 92.4%; specificity: 84.1%
	Deep learning algorithm	Tests differentiated between benign IPMN and malignant IPMN	206 patients with IPMN with confirmed pathology after surgical procedures	Sensitivity: 95.7%; specificity: 92.6%; accuracy: 94.0%, far exceeding the human diagnostic accuracy of 56.0%
Gastrointestinal stromal tumors (GISTs)	AI system based on pathologically histologically confirmed EUS images	Distinguishing between GISTs and GILs	132 of 508 consecutive subjects who were histologically diagnosed	Accuracy increasing from 73.8% to 88.8%
	CNN-assisted diagnostic system	Distinguish GISTs from benign tumors (eg, leiomyomas and schwannoma)	The 587 images of 179 gastric tumors that were used as a training image dataset, and the 212 images of 69 gastric tumors that were used as an independent test dataset.	GIST and non-GIST tumors Sensitivity: 79.0%; Specificity: 78.0%.
				Leiomyomas and Schwannomas Sensitivity: 89.3%; Specificity: 80.6%.
	Diagnosis of subepithelial lesions of the upper gastrointestinal tract	A total of 16,110 images collected from the 631 cases were used in both datasets	Sensitivity: 98.8%; specificity: 67.6%; accuracy: 67.6%; be higher than those of endoscopists
Early esophageal cancer	Cascade region-convolutional neural network model algorithm group	Diagnosis of early esophageal cancer and precancerous lesions	80 patients who met the criteria selected and randomly divided into 3 groups: the traditional CNN model, cascade RCNN model, and EUS alone	Detection rate of the cascade RCNN model: 88.8%
	A CNN algorithm system for identifying the depth of invasion and origin of esophageal lesions	To identify the depth of invasion and lesion origin of esophageal submucosal tumors	1670 endoscopic sonographic images	Accuracy: 82.49%; sensitivity: 80.23%; specificity: 90.56%
Barrett-associated esophageal adenocarcinoma	AI system–assisted ultrasound endoscopy	Staging of the Barrett-associated esophageal adenocarcinoma performed	1020 images of 577 patients with Barrett adenocarcinoma	Overall diagnostic accuracy rate: 73%
Biliary tract lesions	BP MASTER System based on the deep CNN	Real-time identification and bile duct annotation in EUS	2000 ordinary gastroscope images and 2000 EUS images applied	Accuracy rate: DCNN 1: 100%; DCNN 2: 87.4%; DCNN 3: 93.3%; DCNN 4: 77%
	ResNeT50 AI-assisted EUS diagnostic system	Distinguish between neoplastic gallbladder polyps and gallbladder adenocarcinoma	1039 endoscopic images were trained, internal confirmation, and testing, and an external validation cohort of 83 patients validated the diagnostic performance	Accuracy rate comparable to that of endoscopic endoscopists
Focal liver lesions (FLL)	A CNN model based on endoscopic ultrasonography	To identify and classify FLL	A total of 210,685 EUS images from 256 patients that were used to train, validate, and test the CNN model	Random still images Sensitivity: 90%; specificity: 71%
				Assessed full-length video datasets Sensitivity: 100%; specificity: 80%

Although an AI-based computer aid seems promising in the analysis of EUS images of pancreatic lesions, current data need to be interpreted with caution, and the following limitations of machine learning need to be acknowledged^[99]^: the application of AI in EUS is still in its early stage, and several challenges must be addressed to fulfill its potential. One of the major challenges is the need for large amounts of high-quality training data. Developing AI algorithms for endoscopic diagnosis requires large datasets with annotated endoscopic images and clinical data that are time-consuming and costly to acquire. Other challenges are the possibility of overfitting and bias in AI algorithms. Overfitting occurs when an AI algorithm is trained on a dataset that is too small or too homogeneous, and because of that, it performs poorly on new data. Biases occur when AI algorithms are trained on datasets that do not represent the target population and thus may result in inaccurate or unfair results. To address these challenges, future research should focus on developing large, diverse EUS images, and clinical datasets, as well as developing more powerful and transparent AI algorithms. It is also important to note that there are very few prospective studies on AI in the diagnosis or prediction of clinical outcomes and even fewer user-centered algorithms. Artificial intelligence technology can be smoothly embedded in the clinical diagnosis and treatment link only through strict demonstration and research under repeated verification in the real environment to realize a perfect combination of AI and the artificial process

A more serious problem is how to establish a sound AI medical malpractice accountability system. Artificial intelligence technology will undoubtedly change the traditional doctor-patient relationship, and the inherent reason for this change is the potential shift in doctors' personal sense of responsibility. For example, in regard to predicting the nature of digestive tract tumors, misjudgments caused by AI can allow patients to undergo unnecessary surgery or delay treatment. There are multiple sources of accountability: doctors, vendors of software platforms, developers who build algorithms, and training data. The establishment of a perfect accountability system is an important link in the clinical application of digestive endoscopy, but how to divide the responsibility still needs to be clarified.
